# Differential expression of VEGFR2 protein in HER2 positive primary human breast cancer: potential relevance to anti-angiogenic therapies

**DOI:** 10.1186/s12935-017-0427-5

**Published:** 2017-05-19

**Authors:** Aejaz Nasir, Timothy R. Holzer, Mia Chen, Michael Z. Man, Andrew E. Schade

**Affiliations:** 1 Diagnostic and Experimental Pathology, Eli Lilly and Company, Indianapolis, IN USA; 2 Oncology Statistics, Eli Lilly and Company, Indianapolis, IN USA; 3 Eli Lilly and Company, Lilly Corporate Center, DC0424, Indianapolis, IN 46285 USA

**Keywords:** Breast cancer, HER2, Angiogenesis, VEGFR2, Ramucirumab, Antiangiogenic therapy

## Abstract

**Background:**

Clinically relevant predictive biomarkers to tailor anti-angiogenic therapies to breast cancer (BRC) patient subpopulations are an unmet need.

**Methods:**

We analyzed tumor vascular density and VEGFR2 protein expression in various subsets of primary human BRCs (186 females; Mean age: 59 years; range 33–88 years), using a tissue microarray. Discrete VEGFR2+ and CD34+ tumor vessels were manually scored in invasive ductal, lobular, mixed ductal-lobular and colloid (N = 139, 22, 18, 7) BRC cores.

**Results:**

The observed CD34+ and VEGFR2+ tumor vascular counts in individual cases were heterogeneous. Mean CD34+ and VEGFR2+ tumor vessel counts were 11 and 3.4 per tumor TMA core respectively. Eighty-nine of 186 (48%) cases had >10 CD34+ tumor vessels, while 97/186 (52%) had fewer CD34+ vessels in each TMA core. Of 169 analyzable cores in the VEGFR2 stained TMA, 90 (53%) showed 1–5 VEGFR2+ tumor vessels/TMA core, while 42/169 (25%) cores had no detectable VEGFR2+ tumor vessels. Thirteen of 169 (8%) cases also showed tumor cell (cytoplasmic/membrane) expression of VEGFR2. Triple-negative breast cancers (TNBCs) appeared to be less vascular (Mean VD = 9.8, range 0–34) than other breast cancer subtypes. Overall, VEGFR2+ tumor vessel counts were significantly higher in HER2+ as compared to HR+ (p = 0.04) and TNBC (p = 0.02) tissues. Compared to HER2− cases, HER2+ breast cancers had higher VEGFR2+ tumor vessel counts (p = 0.007).

**Conclusion:**

Characterization of pathologic angiogenesis in HER2+ breast cancer provides scientific rationale for future investigation of clinical activity of agents targeting the VEGF/VEGFR2 axis in this clinically aggressive breast cancer subtype.

## Background

Breast cancer is a heterogeneous disease with distinct histopathologic, epidemiologic, clinical, biologic and molecular characteristics. Compared to other solid tumors, human breast cancers exhibit very different clinico-pathologic characteristics and increasingly defined patterns of therapeutic sensitivity and resistance to various targeted therapies. Individualized treatment strategies consider the patient age, performance status, prior therapies and disease stage, but rely primarily on HER2 and hormone receptor status [1].

The proto-oncogene, c-erbB2 encodes the human epidermal growth factor receptor 2 (HER2), which is overexpressed and/or amplified in several human malignancies, including 25–30% of breast cancers [2, 3]. Trastuzumab, a monoclonal antibody directed against the extracellular domain of HER2, is approved for the treatment of HER2-positive breast cancer and improves overall survival [4]. Despite initial efficacy, drug resistance ultimately develops and most tumors progress within 1 year [5]. There is, therefore, still an unmet clinical need to improve patient outcome in trastuzumab-treated BRC patients.

The introduction of anti-angiogenic (AA) therapies represents a major advancement in treating human cancers. Despite favorable clinical trial results and several regulatory approvals (Table 1), majority of patients who initially respond to anti-angiogenic therapies eventually develop progressive disease [6]. Furthermore, the duration of improved patient survival remains modest and needs to be improved. De novo or acquired resistance to anti-angiogenic therapies is another major clinical challenge.

**Table 1:** FDA approved drugs targeting VEGF/VEGFR pathways

Name	Company	Type	Main target(s)	Approved for
Bevacizumab (Avastin)	Genentech/Roche	Humanized monoclonal antibody	VEGF-A	Metastatic colorectal carcinoma, non-small cell lung carcinoma, advanced glioblastoma, metastatic renal cell carcinoma
Sorafenib (Nexavar)	Bayer/Onyx	Small molecule TK inhibitor	VEGFR, PDGFR, Raf, cKit, FLT3	Advanced renal cell and hepatocellular carcinomas
Axitinib (Inlyta)	Pfizer	Small molecule TK inhibitor	VEGFR, PDGFR, cKit	Renal cell carcinoma
Pazopanib (Votrient)	GSK	Small molecule TK inhibitor	VEGFR, PDGFR, cKit	Advanced renal cell carcinoma, soft tissue sarcoma
Vandetanib (Caprelsa)	AstraZeneca	Small molecule TK inhibitor	VEGFR, EGFR, RET	Medullary thyroid cancer
Zaltrap (VEGF trap)	Regeneron/Sanofi-aventis	Fusion protein of Fc with VEGFR1 and R2 domains	VEGF, PlGF	Metastatic colorectal cancer
Ramucirumab (Cyramza)	Lilly/Imclone	Human monoclonal antibody	VEGFR2	Gastric/gastroesophageal junction adenocarcinoma, colorectal carcinoma, non-small cell lung carcinoma

A role of vascular endothelial growth factor (VEGF) in breast cancer progression is supported by clinical studies showing elevated serum VEGF levels in invasive breast cancers [7]. However, the aggregate outcomes of a number of positive randomized phase III clinical trials evaluating the VEGF-pathway inhibitor (bevacizumab) or the antiangiogenic tyrosine kinase inhibitors (TKIs), with or without concurrent chemotherapy, in metastatic breast cancer patients have been disappointingly modest or negative [8, 9]. More recently, therapeutic blockade of VEGFR2 with the human anti-VEGFR2 monoclonal antibody (ramucirumab), which, based on successful phase III trials [10–12], was approved by the FDA for gastric, non-small cell lung and colon cancers, but did not meaningfully improve important clinical outcomes in a randomized placebo-controlled phase III trial evaluating the addition of ramucirumab to first-line docetaxel chemotherapy in metastatic breast cancer [13].

In order to address the important clinical challenges with the antiangiogenic therapies in breast cancer patients, there is an urgent need to develop clinically applicable predictive biomarkers to tailor various AA therapies to the most relevant BRC patient subpopulations. Other strategies to improve efficacy of AA therapies in BRC patients would include evaluation of various BRC subtypes for expression of pertinent biomarkers of pathologic angiogenesis (disease state characterization) and combining AA-agents with other established (hormonal, anti-HER2) or emerging targeted therapies.

With the ultimate objective to de-convolute the biologic complexity underlying some of the clinical challenges with the AA therapies outlined above, we have developed and standardized technically robust immunohistochemical assays to evaluate VEGF receptor pathway markers in archival human cancer tissues. In recent years, these methodologies have been utilized to characterize the heterogeneity of tumor angiogenesis programs in various histologic and clinical subtypes of human cancers [14–19]. Previously, we demonstrated immunohistochemical expression of VEGFR2 protein and vascular phenotypes in human breast carcinomas [15]. The aim of this study was to characterize the patterns of pathologic angiogenesis in various therapeutically relevant molecular breast cancer subtypes (HR+, HER2+, TNBC) by evaluation of vascular density and immunohistochemical expression of VEGFR2 protein in a retrospective series of primary human breast cancer tissues.

## Methods

In line with the original REMARK guidelines [20] to standardize reporting of tumor marker studies, the study patients, tissue specimens, methodologies, including reagents, controls and various other parameters are being summarized.

### Patients

The study population included a retrospective series of 186 female patients, including 171 Caucasian, 11 African American and 4 others with node-positive primary breast cancers (89 left, 90 right and 5 bilateral). Mean patient age was 59 years (range 33–88 years). Most of these patients received local radiation and chemotherapy, including Adriamycin, Cytoxan and 5-FU, as previously described [21]. Clinico-pathologic data were collated from the Yale Tumor Registry in accordance with the guidelines of the Yale Human Investigations Committee.

### Human Tissue specimens and tissue microarray

Mean primary breast cancer size was 3.4 cm (range 0.15–14.5 cm). Primary human breast carcinoma tissues (N = 186) were classified into invasive ductal, lobular, mixed ductal-lobular and mucinous carcinomas (N = 139, 22, 18, 7 respectively), based on the original pathologic evaluation at Yale University. Using the Nottingham Modification of the Scarff-Bloom-Richardson grading system, also known as the Nottingham Grading System (NGS) [22], the invasive carcinoma tissues (N = 186) were categorized into grade 1 (N = 8), grade 2 (N = 109), grade 3 (N = 69). Representative formalin-fixed, paraffin-embedded (FFPE) tumor tissue from each case was sampled as a single 0.6 mm core in a recipient tissue microarray block (Yale BRCA, YTMA 10), on a tissue-arraying instrument (Beecher Instruments, Silver Springs, MD). Sampling of human tissues in the Yale BRC TMA was based on the required institutional policies and approvals, including the patient consent to allow usage of tissue for research. Using the latest criteria proposed by the World Health Organization for Histologic Typing of Breast Tumors [23], all original pathologic diagnoses were confirmed on Hematoxylin & Eosin stained section of the Yale BRCA TMA (YTMA 10) by an experienced American Board-certified study pathologist (AN) with subspecialty expertise in breast pathology.

### Immunohistochemical assays for VEGFR2 and CD34

Five micron thick FFPE TMA sections were cut from the Yale Breast Cancer TMA above, stored in nitrogen chamber to prevent loss of antigenicity until immunostained for VEGFR2 and CD34, a sensitive IHC marker for tumor vasculature. For VEGFR2 protein we used a technically robust, sensitive, specific and selective immunohistochemical (IHC) assay developed and optimized by our laboratory (13) that had showed optimal performance on several different human tumor cohorts, including multiple human tissue and cell lines controls (14–17, 19). The IHC assay protocol, using one of the most specific commercially available monoclonal anti-VEGFR2 antibody (55B11) [24], optimization experiments and quality control procedures were previously described in detail [14]. For CD34, we used a technically validated IHC assay offered by a leading reference laboratory (Clarient, Aliso Viejo, CA, USA), including satisfactory positive and negative controls.

### VEGFR2 IHC assay controls

Unequivocal, crisp VEGFR2 immunoreactivity was demonstrated in the vascular endothelium but not in trophoblastic cells in the conventional sections of human placenta and also in the microvasculature of the invasive cervical squamous cell carcinoma favoring these tissues as optimal positive and negative tissue controls. Optimal reagent negative controls were run by replacing the primary antibody with control immunoglobulin.

### VEGFR2 IHC assay performance

As part of the analytical validation of the VEGFR2 IHC assay, the coefficients of variation (CVs) of immuno-pathological VEGFR2 scores for intra-run repeatability, inter-run reproducibility, and inter-observer reproducibility were less than 7% (data on file).

### Interpretation and scoring of VEGFR2+ and CD34+ tumor vessels

After immuno-pathologic review, each immuno-stained BRC TMA section was evaluated by the sub-specialty pathologist (AN), who was blinded to the BRC subtypes or other relevant clinico-pathologic or breast marker data. In each analyzable TMA core, the discrete VEGFR2+ and CD34+ tumor vessels were manually counted in the invasive tumor stroma. In order to qualify for a vascular structure, it had to have the histomorphologic appearance of a vessel with or without lumen. Scattered individual cells in the invasive cancer tissue stroma not conforming to the strict definition of a vessel above were excluded from VEGFR2+ and CD34+ vessel counts. Any suboptimal/inadequate TMA cores (cores with complete or major [>50%] tissue loss/fragmentation; those without well-preserved, viable tumor cells or those with any areas of tumor necrosis) were also excluded from scoring/analysis. After such exclusions, a total of 186 and 169 TMA cores were found to be adequate for manual assessment of CD34+ and VEGFR2+ vessels in the stromal component of the primary invasive carcinoma tissues sampled (Table 2).

**Table 2:** Distribution of frequency of CD34+ and VEGFR2+ vessels in human breast cancer stroma (all histologic subtypes)

No. of marker+ tumor stromal vessels/TMA core	0	1–5	6–10	>10	Mean # vessels/TMA core (range)	Total number of evaluable cores
CD34+ tumor vessels	9	36	52	89	11 (0–45)	186
VEGFR2+ tumor vessels	42	90	29	8	3.4 (0–20)	169

About half of the evaluable TMA cores exhibited more than 10 CD34+ vessels in the tumor stroma. About half the evaluable TMA cores had lower number (1–5) of VEGFR2+ vessels/TMA core, while about a quarter of cases had no VEGFR2 expressing vessels in the tumor stroma

### Photomicrography

Photomicrographic images representing the differential levels of immunohistochemical expression of VEGFR2 in tumor stromal vasculature in HR+, HER2+ and triple-negative BRCs were captured from high-resolution digital scans of the stained TMA slides (Scanscope XT; Aperio Technologies, Vista, CA).

### Relevant immunohistochemical markers: ER, PR, HER2

Based on the breast marker IHC panel results (ER, PR and HER2) from the contributing institution (Yale University, New Haven, CT, USA), as used in the standard management of breast cancer patients, each case was grouped into one of the three BRC subsets, i.e., hormone receptor+ (HR+), HER2+ and triple-negative (TNBC).

### Statistical methods

Counts of VEGFR2+ tumor vessels from 3 breast cancer subtypes (HR+, HER2+, and TNBC) were compared for all pairs using Tukey–Kramer HSD procedure (JMP 12.1.0, SAS Institute Inc.). Because the distribution of the data was heavy-tailed to the right, various transformations were tried to normalize the data. However, none of the transformations normalized the data satisfactorily. Hence, zero-inflated Poisson regression was used to account for excessive zeroes in the data [25, 26]. Counts of VEGFR2+ tumor vessels from 2 aggregated breast cancer subtypes (HER2+ vs. HER2−) were compared using t test and zero-inflated Poisson regression.

## Results

After exclusion of suboptimal/inadequate cores from the stained TMA sections as outlined above, a total of 164 cases had both CD34+ and VEGFR2+ tumor stromal vessel counts from the same TMA cores for comparison. Among those, 98 (60%) were HR+, 20 (12%) were HER2+ and 46 (28%) were triple-negative.

### Immunohistochemical localization of CD34+ and VEGFR2+ vessels in breast cancer stroma

The observed CD34+ and VEGFR2+ tumor vascular counts in individual breast cancer cases were heterogeneous. Overall, the BRC cases analyzed had larger numbers of CD34+ tumor stromal vessels per TMA core (mean 11; range 0–45) as compared to VEGFR2+ tumor stromal vessels per TMA core (mean 3.4; range 0–20). Also, 89 of 186 evaluable TMA cores exhibited more than 10 CD34+ vessels/core, while only 8 of 169 evaluable TMA cores had more than 10 VEGFR2+ vessels/core (Table 2), implying that only a proportion of CD34+ tumor stromal vessels co-expressed VEGFR2 protein in their endothelial lining.

### Vascular expression of VEGFR2 in various breast cancer subtypes

**Fig. 1:**
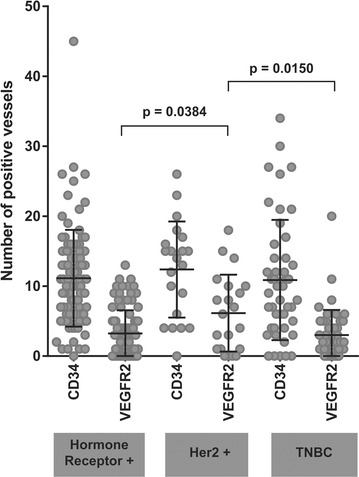
Scatter dot plot showing individual BRC cases (*grey symbols*) representing the number of tumor stromal vessels showing unequivocal immunoreactivity for CD34 and VEGFR2 in various BRC subsets. Means and SD are represented by *black lines* both for CD34 and VEGFR2. Comparisons between VEGFR2 positive vessel counts in HER2+ vs. HR+ BRCs and HER2+ BRCs vs. TNBCs are shown with statistically significant results marked with an *asterisk* (T test)

**Fig. 2:**
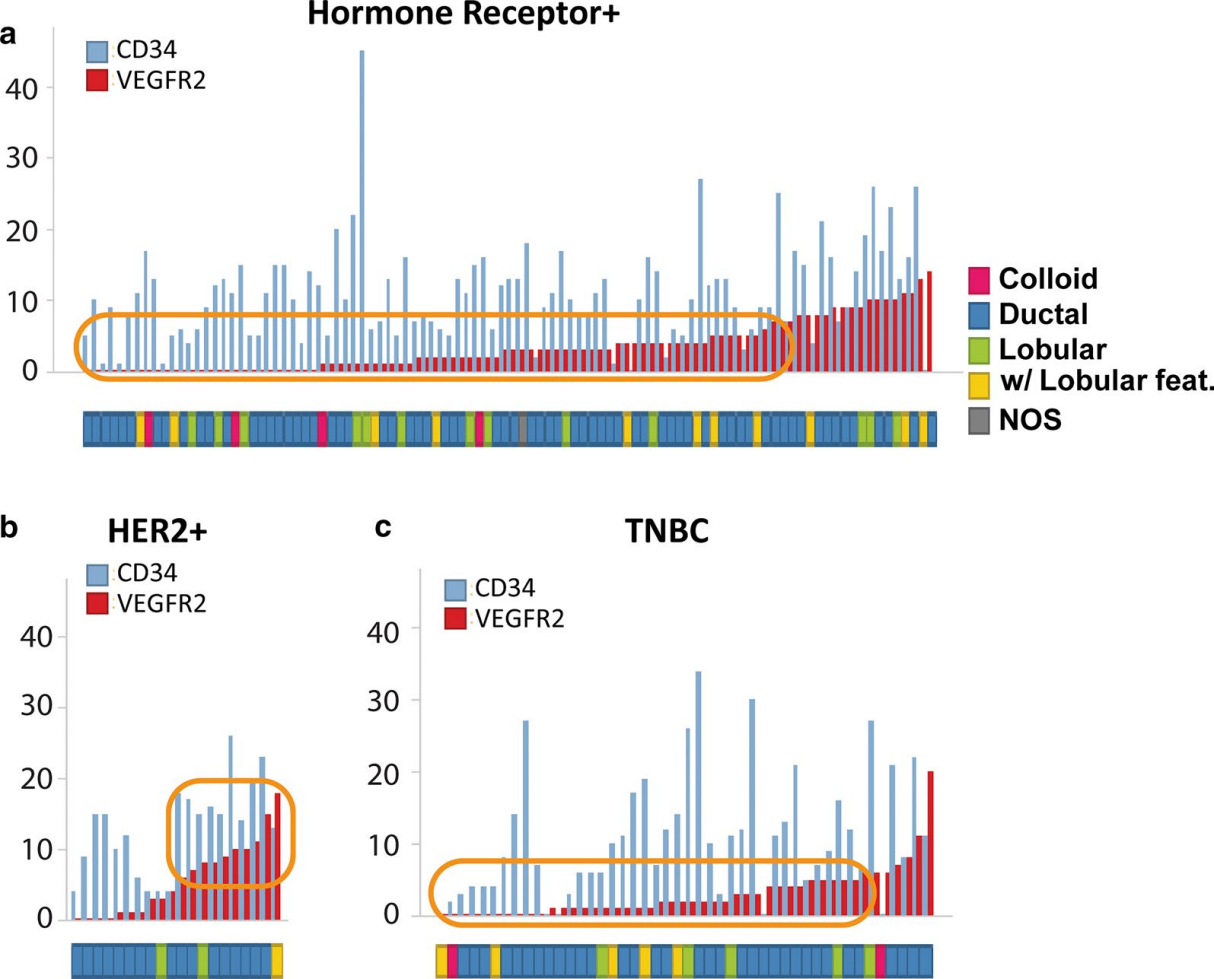
**a**–**c** CD34+ and VEGFR2+ vascular counts in various human breast cancer subtypes. Within each BRC subtype, cases were arranged from negative to low (1–5) to intermediate (6–10) to high (>10) VEGFR2+ vessel counts. As compared to hormone receptor positive (**a**) and triple negative (**c**) BRCs, a greater proportion of HER2+ (**b**) BRCs exhibited higher numbers of VEGFR2+ vessels in the tumor stroma (*orange boxes*)

**Fig. 3:**
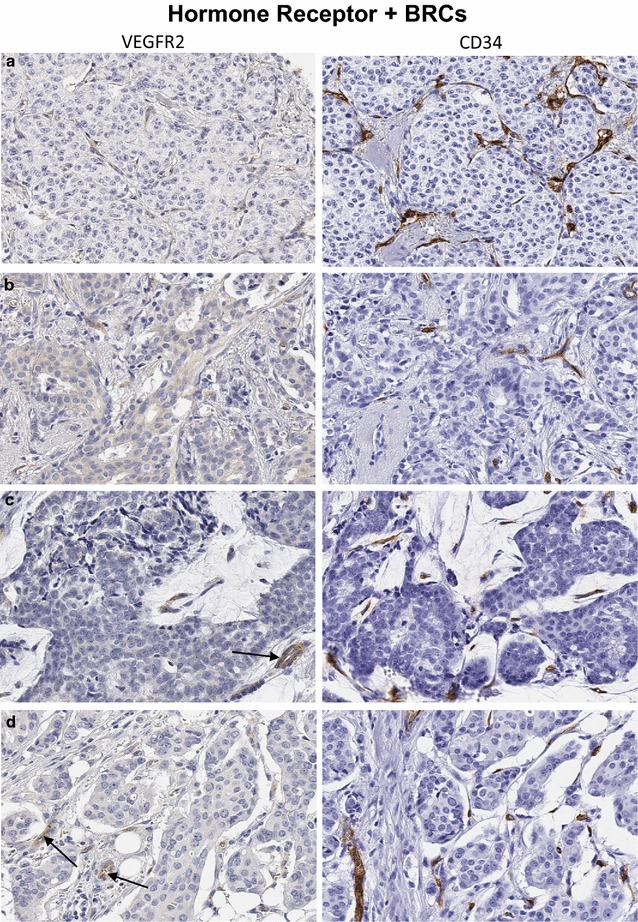
**a**–**d** Invasive carcinomas of the breast (hormone receptor+) representative of the majority of cases in Fig. 2a (*orange box*). While there are a number of CD34+ stromal vessels in each case (*right panels*), only an occasional tumor stromal vessel shows immunoreactivity for VEGFR2 (*left panels*). **a** Invasive lobular carcinoma without obvious VEGFR2+ vessels in tumor stroma. **b** Invasive ductal carcinoma. **c** Mucinous carcinoma with a mixture of VEGFR2+ (*black arrow*) and VEGFR2− vessels in the tumor stroma. **d** Invasive lobular carcinoma with an occasional VEGFR2+ vessel in the tumor stroma. Original magnification ×200

**Fig. 4:**
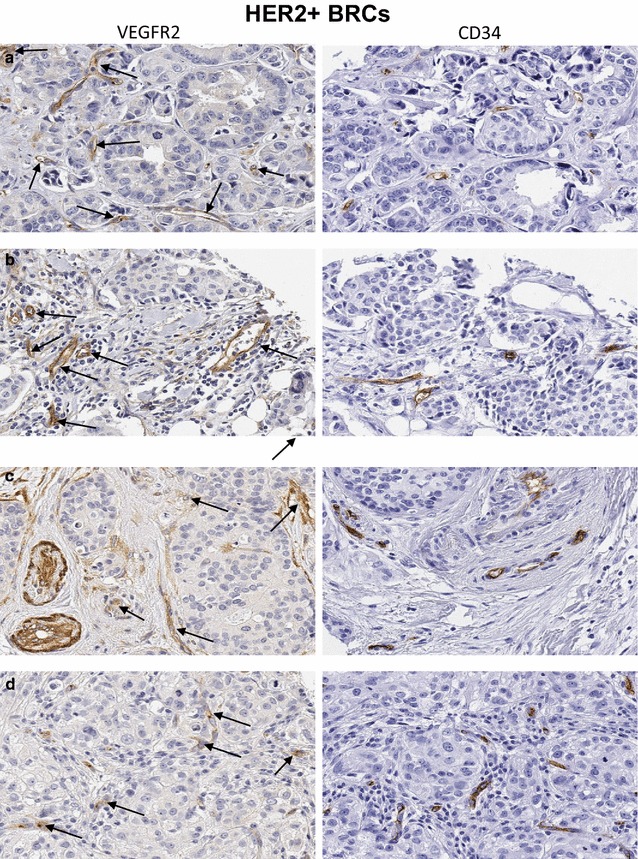
**a**–**d** Invasive carcinomas of the breast (HER2+) representative of the significant proportion of cases in Fig. 2b (*orange box*). Overall, these cases show significantly higher numbers of VEGFR2+ tumor vessel counts and crisp brown staining for VEGFR2 protein in majority of the tumor stromal vessels (*left panels*, *black arrows*), despite variable numbers of CD34+ tumor stromal vascular counts (*right panels*). **a** Invasive ductal carcinoma. **b** Invasive breast carcinoma with lobular features. **c** Invasive ductal carcinoma. **d** Invasive lobular carcinoma. Original magnification ×200

**Fig. 5:**
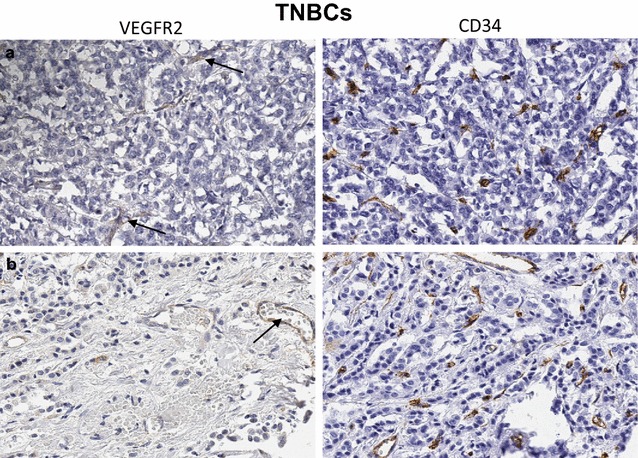
**a**, **b** Invasive carcinomas of the breast, triple-negative (TNBCs), representative of the majority of cases in Fig. 2c (*orange box*). Invasive ductal (**a**) and invasive lobular (**b**) carcinomas of the breast, featuring fairly high CD34+ tumor vessel counts (*right panels*). In both cases only a rare tumor stromal vessel shows immunoreactivity for VEGFR2 (*black arrows*, *left panels*). Original magnification ×200

Overall, the levels of vascular expression of VEGFR2 were relatively low in histologically characterized breast cancer tissues. Of 164 breast cancer cases including all histologic types, 127 (77.4%) had a few (<5) or no VEGFR2 positive tumor vessels in the tissue sampled in the TMA cores (Table 3), while 37 (22.4%) showed intermediate or high vascular expression of VEGFR2. among the various molecular BRC subtypes, however, VEGFR2+ tumor stromal vessel counts were significantly higher in HER2+ (mean 6.1 [sd 5.5], median 6) as compared to HR+ (mean 3.2 [3.3], median 3, p = 0.04) and triple negative BRCs (mean 3.0 [3.6], median 2, p = 0.02) tissues (Figs. 1, 2, 3, 4, 5). There was no significant difference between VEGFR2+ tumor stromal vessel counts between HR+ and triple negative BRCs (p = 0.69). As compared to HER2+ breast cancer cases illustrated in Fig. 4, in which many of the tumor stromal vessels localized by CD34 immunoreactivity (a–d; right panels) were also VEGFR2+ (a–d; left panels), the HR+ breast cancers illustrated in Fig. 3, despite showing frequent localization of CD34+ tumor stromal vessels (a–d; right panels), only showed an occasional VEGFR2+ vessel in the tumor stroma (a–d; left panels, black arrows). Similarly, compared to HER2+ breast cancer cases illustrated in Fig. 4, in which many of the tumor stromal vessels localized by CD34 immunoreactivity (a–d; right panels) were also VEGFR2+ (a–d; left panels), the TNBCs illustrated in Fig. 5, despite showing frequent localization of CD34+ tumor stromal vessels (a, b; right panels), only showed an occasional VEGFR2+ vessel in the tumor stroma (a, b; left panels, black arrows).

**Table 3:** Immunohistochemical expression of VEGFR2 protein in stromal vessels among various histologic subtypes of human breast cancer

Histology	Cases (#)	VEGFR2+ tumor vessels/TMA core	VEGFR2+ tumor vessels/TMA core	VEGFR2-neg to low	VEGFR2-intermediate	VEGFR2-high
		Mean (sd)	Median	No. of cases (%)	No. of cases (%)	No. of cases (%)
Ductal	120	3.5 (3.8)	3	93 (77.5)	21 (17.5)	6 (5)
Lobular	20	3.5 (3.4)	2.5	15 (75.0)	5 (25.0)	0
Mixed	18	4.3 (5.1)	2.5	14 (77.8)	1 (5.5)	3 (16.7)
Mucinous	6	1.5 (2.4)	0.5	5 (83.3)	1 (16.7)	0
Total	164			127 (77.4)	28 (17.0)	9 (5.4)

Based on CD34+ and VEGFR2+ vascular counts in various human breast cancer subtypes, cases were ranked from negative to low (1–5) to intermediate (6–10) to high (>10) VEGFR2+ vessel counts (Fig. 2). Compared to HR+ BRCs and TNBCs, a greater proportion of HER2+ BRC cores had higher numbers of VEGFR2+ tumor vessels (Fig. 2a–c). Also, compared to HER2-negative BRCs, HER2+ BRCs had significantly higher VEGFR2+ tumor vessels count (p = 0.007). In mucinous carcinoma, weak VEGFR2 staining was found in an occasional tumor stromal vessel.

### Tumor cell expression of VEGFR2 protein

Thirteen of 169 (8%) cases also showed tumor cell (cytoplasmic and/or membrane) expression of VEGFR2 protein.

## Discussion

Most clinical trials of AA-agents in BRC have reported improved response rate and PFS but no increase in OS compared to chemotherapy alone [9]. In metastatic breast cancer patients, clinical outcomes of a number of positive randomized phase III clinical trials evaluating the VEGF-pathway targeted therapies, with or without concurrent chemotherapy, have been disappointingly modest [8]. Furthermore, in the last few years, the reported association of HER2+ BRC and angiogenesis has been leveraged in a number of clinical trials, in which various combinations of trastuzumab, lapatinib, and bevacizumab have shown increased efficacy and that combined anti-HER2 and anti-VEGF treatment may overcome resistance to anti-HER2 monotherapy [27–29]. In a phase III trial [30], combination of bevacizumab, docetaxel and trastuzumab failed to improve progression‐free survival in locally recurrent/metastatic BRC patients.

An association between HER2 signaling and angiogenesis is suggested by several lines of evidence: (1) overexpression of HER2 in human tumor cells is closely associated with increased angiogenesis and expression of VEGF [3, 31]; (2) ErbB2 increases VEGF protein synthesis via activation of mTOR/p70S6K pathway leading to increased angiogenesis and spontaneous metastasis of human breast cancer cells [32]; (3) expression of VEGF-A, VEGF-C and VEGF-D was significantly and positively correlated with ErbB2 expression in human BRC [33]; (4) The positive association between HER-2 and VEGF expression implicates VEGF in the aggressive phenotype exhibited by HER-2 overexpression, and supports the use of combination therapies directed against both HER-2 and VEGF for HER2 overexpressing BRCs [34]; (5) in experimental models, combined trastuzumab plus paclitaxel treatment inhibited HER2-mediated angiogenesis along with tumoricidal effects via the reduction of phospho-Akt [35]; (6) HER2 signaling increases the rate of hypoxia-inducible factor 1alpha (HIF-1alpha) synthesis, which in turn mediates VEGF expression [36]; (7) HER2 signaling modulates the equilibrium between pro- and antiangiogenic factors via distinct pathways with potential implications for HER2-targeted antibody therapy [37]. Therefore, novel therapeutic approaches to overcome primary and secondary resistance to trastuzumab include inhibition of angiogenesis and other signaling pathways (PI3K/mTOR, IGF1-R, HSP90) involved in breast cancer growth [5]. These are further supported by preclinical studies that suggest potential for increased efficacy with combined inhibition of HER2 and VEGF pathways [28, 38–40].

The introduction of AA therapies represents a major advancement in treating human cancers. Despite favorable clinical trial results and several regulatory approvals, a majority of patients who initially respond to anti-angiogenic therapies, eventually develop progressive disease. Furthermore, the duration of improved patient survival remains modest and needs to be improved. De novo or acquired resistance to anti-angiogenic therapies is another major clinical challenge.

While scientific rationale to treat HER2‐overexpressing BRC with anti‐angiogenic therapy exists, treating unselected HER2+ BRC patient populations with anti‐angiogenic therapies without reliable predictive biomarkers continues to be a major clinical challenge. In order to investigate this important challenge, we used a technically sound IHC assay for VEGFR2 protein developed in our lab [14], and analyzed a large well-characterized primary breast cancer tissue cohort including various BRC subtypes (HR+, HER2+, TNBC). For each case, immunohistochemical expression of VEGFR2 protein was evaluated in the tumor vasculature outlined by vascular endothelial immunoreactivity for CD34.

Taking all histologic/molecular subtypes of BRCs together, the observed levels of vascular expression of VEGFR2 were relatively low. The majority of HR+ BRCs and TNBC tissues analyzed had only a few or no VEGFR2 positive tumor stromal vessels in the respective TMA cores. Since VEGFR2 is the main receptor that mediates VEGF/VEGFR2 signaling, sporadic vascular expression of VEGFR2 in the majority of BRC tissues analyzed along with the failure of clinical trials of anti-angiogenic agents [13, 41, 42] to show clinical benefit in unselected BRC patient populations, underscores the need to tailor AA-therapies to respective BRC patient sub-populations. This may require administration of biologically relevant AA-therapeutic combinations to achieve higher levels of success in future clinical trials.

Interestingly, among the various BRC subtypes analyzed, we found significantly higher expression of VEGFR2 protein in the tumor stromal vasculature in HER2+ BRCs as compared to HR+ and TNBCs. Similarly, compared to HER2-negative BRCs, HER2+ BRCs had significantly higher expression of VEGFR2 protein in tumor vasculature. Despite higher levels of vascular VEGFR2 expression in tumor vasculature in HER2+ BRCs, overall, only a smaller proportion (8%) of BRC tissues in this analysis showed cytoplasmic and/or membrane expression of VEGFR2 protein in invasive carcinoma cells. Predominance of vascular expression of VEGFR2 in the present study is in line with some earlier studies [43, 44], but in contrast to others [45–47], in which frequent expression of VEGFR2 has been reported in infiltrating BRC cells. This is also in contrast to a prior study from our laboratory on non-small cell lung carcinoma tissues, in which VEGFR2 expression was a more frequent finding both in pulmonary adenocarcinomas and squamous cell carcinomas [14] and far more frequent tumor cell expression of VEGFR2 protein in squamous cell carcinomas from other sites like cervix and head and neck (unpublished observations). Considering the major role of VEGFR2 in VEGF-induced angiogenesis in human cancer, the finding of high VEGFR2 expression in HER2+ BRC provides scientific rationale to study clinical activity of therapeutic blockade of VEGFR2 in this clinically aggressive breast cancer subtype.

In invasive BRCs, VEGFR2 (Flk-1/KDR) expression shows significant correlation with proliferation indices like Ki-67 and topoisomerase-II alpha, implying that VEGF may exert a growth factor activity on BRC cells through its receptor (VEGFR2) [48]. Recently, cyclin D1/CDK4 is shown to mediate targeted therapy resistance in HER2+ breast cancer [49], while CDK4/6 inhibition reduces TSC2 phosphorylation, mTORC1 activity and cell proliferation, increases tumor cell dependence on EGFR family kinase signaling [50] and provides a potent adjunct to HER2-targeted therapies in preclinical breast cancer models [51]. Since CDK4/6 inhibitors re-sensitize PDX tumors to HER2-targeted therapies and delay tumor recurrence in vivo, CDK4/6 inhibitors may also re-sensitize resistant HER2+ human BRCs to EGFR/HER2 inhibition [50].

Although our analyses were carried out on a well-characterized BRC cohort using technically robust IHC assays with optimal controls, relative limitations of this study include available sample size and the use of BRC tissue microarray rather than whole tumor tissue sections. While the use of TMA technology is well established in evaluation of novel tissue biomarkers as an efficient and cost-effective approach, it can potentially contribute to overestimation or underestimation of biomarker expression as well. Given that the overall IHC expression of VEGFR2 in the BRCs tissues analyzed in the present study was relatively low, the observed differences in VEGFR2 expression in HER2+ and other BRC subsets may in part be due to heterogeneity of VEGFR2 expression in the TMA cores evaluated. However, in the context of multiple lines of scientific evidence summarized above, supporting increased tumor angiogenesis in HER2+ BRC, the observed differences in vascular VEGFR2 expression in various BRC subtypes are suggestive of underlying biology. Some of the sampling related questions may be addressed by further evaluation and verification of these observations in independent analyses of larger series of well-characterized HER2+ and other subsets of human breast cancer tissues, using conventional tumor sections and other molecular methodologies.

Since therapeutic targeting of HER2 or VEGF alone does not provide adequate tumor control in many of the treated patients [52, 53], evaluation of newer targeted approaches with or without other anti-HER2 therapies may be relevant to inhibit pathologic angiogenesis in HER2+ breast cancer.

## Conclusion

Using a technically robust immunohistochemical assay developed in our laboratory, our disease state characterization analyses have demonstrated significantly higher expression of VEGFR2 protein in HER2+ breast cancer compared to other BRC subtypes. Based on these findings, we hypothesize that compared to hormone receptor positive or triple negative subsets, HER2+ human breast cancers with high VEGFR2 expression may respond differently to anti-angiogenic therapies. While these data are provocative in providing biologic insight into the pathologic angiogenesis program in human HER2+ BRC, these findings merit further investigation and independent validation.

## Abbreviations

5-FU: 5-FluoroUracil
AA therapy: anti-angiogenic therapy
BRC: breast cancer
CDK4/6: cyclin dependent kinases 4/6
EGFR: epidermal growth factor receptor
ER: estrogen receptor
FDA: Food and Drug Administration (USA)
FFPE: formalin fixed paraffin embedded
HER2: Human Epidermal Growth Receptor 2
HR+: hormone receptor positive
IHC: immunohistochemistry
KDR: kinase domain receptor (Synonym for VEGFR2)
mTOR: mammalian target of rapamycin
NGS: Nottingham Grading System
OS: overall survival
PDX: patient derived xenografts
PFS: progression free survival
PR: progesterone receptor
REMARK: REporting recommendations for tumour MARKer prognostic studies
TKIs: tyrosine kinase inhibitors
TMA: tissue microarray
TNBC: triple negative breast cancer
VEGF: vascular endothelial growth factor
VEGFR2: vascular endothelial growth factor receptor 2
YTMA: yale tissue microarray
